# Influence of Age and Phylogenetic Background on Blood Parameters Associated With Bone Metabolism in Laying Hens

**DOI:** 10.3389/fphys.2021.678054

**Published:** 2021-04-29

**Authors:** Christin Habig, Annett Weigend, Ulrich Baulain, Stefanie Petow, Steffen Weigend

**Affiliations:** ^1^Institute of Farm Animal Genetics, Friedrich-Loeffler-Institut, Neustadt, Germany; ^2^Institute of Animal Welfare and Animal Husbandry, Friedrich-Loeffler-Institut, Celle, Germany

**Keywords:** diurnal rhythm, egg laying cycle, laying period, bone marker, calcium

## Abstract

The high laying performance of today’s laying hens places enormous demands on their mineral metabolism. While up-to-date data are rare, the present study aimed to describe blood parameters associated with egg laying and bone metabolism during the pre-laying period, in the course of the laying period and the daily egg laying cycle. Ten to 15 laying hens of two high-performing, phylogenetically divergent lines (BLA: brown-egg layer; WLA: white-egg layer), kept in single cages were blood sampled at 17, 25, 29, 49, and 69 weeks of age. Sampling was made at 6 a.m., 10 a.m., 2 p.m. and, with the exception of week 17, 6 p.m. Blood samples were analyzed for concentrations of total and ionized calcium, inorganic phosphate (PO4), markers of bone formation (osteocalcin) and resorption [carboxyterminal crosslinked telopeptide of type I collagen (CTX-I)], 25-hydroxycholecalciferol (25(OH)D_3_) and estradiol-17β. In the pre-laying period (17 week), the estradiol-17β level calculated for WLA was more than twice as high as the level calculated for BLA, while no significant difference could be observed in the laying period (25 to 69 weeks). BLA hens had significantly higher total calcium concentrations at 49 weeks of age as well as up to twice as high levels of osteocalcin and 25(OH)D_3_ than WLA at any time of the day from 25 to 69 weeks of age. While osteocalcin, CTX-I and 25(OH)D_3_ concentrations were significantly higher before the onset of lay, total calcium and estradiol-17β levels significantly increased from 17 to 69 weeks of age. In contrast, PO4 values varied only slightly during the experimental period and ionized calcium was highest at 17 and 49 weeks of age and lowest around peak production (29 week). In the course of the daily egg laying cycle blood concentrations clearly reflected the stage of egg formation. Our results provide up-to-date data of bone- and egg laying-associated blood parameters of two contemporary purebred layer lines over the course of the pre- and egg-laying period and the daily egg laying cycle. Differences between brown- and white-egg layers raise questions, whether phylogenetic background determines their efficiency to cope with high calcium demands relating to egg production.

## Introduction

Decades of breeding aimed at increasing the genetic potential of laying hens to achieve maximum saleable eggs, and simultaneously maximizing cost-effectiveness (Icken et al., 2012). Daily eggshell formation is known as one of the most rapid mineralisation processes (Nys and Le Roy, 2018) and places high demands on hens’ mineral metabolism. Approximately 2.2 g of calcium are required to form the shell of an egg (Bain et al., 2016), which is more than 10% of the total body calcium (Gerstberger and Barth, 2005). Only about 50% of this calcium is covered by intestinal reabsorption from hen’s diet (Kerschnitzki et al., 2014). About one third of the calcium needed for eggshell formation originates from mobilisation from medullary bone (Bouvarel et al., 2011). Medullary bone is a woven bone formed by osteoblasts in female birds when they first come into lay under the influence of estrogen (Bouvarel et al., 2011). It is primarily build in the marrow of long bones (especially femur and tibia) (van de Velde et al., 1984) and serves as a labile source of calcium for the formation of the eggshell (Whitehead, 2004) representing about 12% of total bone calcium (Nys and Le Roy, 2018). Alongside medullary bone resorption, structural bone is also resorbed, leading to osteoporosis (Whitehead, 2004). Consequently a high number of laying hens are supposed to suffer from bone fractures (Gregory and Wilkins, 1989) and keel bone deviations due to a high laying rate (Rufener and Makagon, 2020), although this has recently been questioned as well (Jansen et al., 2020a; Dunn et al., 2021).

The egg-laying cycle of modern laying hens (Bain et al., 2016) and quails lasts about 24 h and can be divided into an active period, where eggshell formation takes place and an inactive period when no eggshell is formed (Miller, 1977). Five hours after ovulation, the ovum reaches the uterus and eggshell formation occurs between 7 and 20 h after ovulation, followed by oviposition 24 h after ovulation (van de Velde et al., 1984). As modern layer lines lay their eggs within a very narrow time frame at the early morning, the final stages of shell formation are at night when hens do not eat and need to release calcium from medullary bone (Bain et al., 2016). Consequently, medullary bone absorption and renewal occur rapidly (Bain et al., 2016) on a daily basis (Riczu et al., 2004).

Two types of cells, namely osteoblasts and osteoclasts are involved in bone metabolism. Osteoblasts are responsible for bone formation by depositing an organic matrix of collagen and non-collagenous proteins and lipids, which mineralizes thereafter (Kerschnitzki et al., 2014). One of these non-collagenous proteins is osteocalcin, released into the blood stream during bone turnover and used as a common marker of bone formation (Seibel, 2005; Bain et al., 2016). By contrast, the carboxyterminal crosslinked telopeptide of type I collagen (CTX-I), a collagen I degradation product, is widely used as a marker of bone resorption. CTX-I is a highly specific bone marker because osteoclasts are only responsible for type I collagen degradation in bone, but not in other tissues (Christenson, 1997). When bone resorption takes place to fulfill calcium requirements during eggshell calcification, concentrations of plasma phosphate also increase (Hurwitz and Bar, 1965) due to the fact, that almost all of the calcium stored in bone is in the form of hydroxyapatite crystals of calcium phosphate (Whitehead and Fleming, 2000).

Regulation of egg formation and bone metabolism underlies a number of factors. Egg formation is controlled by hormones produced from the ovary and pituitary gland, which interact closely and affect the calcium-regulating hormones and organs (Nys and Le Roy, 2018). One of the major hormones that play a role in Ca regulation in birds is estradiol-17β (reviewed by Beck and Hansen, 2004). Estrogens, including estradiol-17β are primarily produced by prehierarchal follicles and 6–3 h before ovulation their secretion from the four largest follicles increases (Johnson, 2000). They are required for activation of the 25(OH)_2_-1-*α*-hydroxylase in the kidney through up-regulation of parathyroid hormone (PTH) receptors. This enzyme converts 25-hydroxycholecalciferol (25(OH)D_3_) to 1,25-dihydroxycholecalciferol (1,25(OH)D_3_), which controls calcium absorption in the gut. Moreover, estrogen up-regulates 1,25(OH)D_3_ receptors located in gut mucosa, has a direct effect on calcium homeostasis in the avian kidney by increasing its responsiveness to PTH and triggers medullary bone production (Pfeiffer and Gardner, 1938).

The majority of existing data regarding changes of blood parameters during the ovulatory cycle are based on investigations made decades ago. However, the breeding progress over the past years was enormous and created laying hens with a high potential of egg production over a prolonged period (Preisinger, 2018). Since white and brown egg-laying chicken lines have different breeding histories and evolved separately (Malomane et al., 2019), phylogenetic origin may also have an influence on the expression of physiological regulatory mechanisms.

The current study aimed to examine several blood parameters associated with egg laying and bone metabolism during the pre-laying period as well as in the course of the laying period and the daily egg laying cycle. We examined two high performing, purebred layer lines differing in phylogenetic background, i.e., with white and brown eggshell color, from 17 to 69 weeks of age.

## Materials and Methods

### Ethical Note

For the current study it was necessary to know exactly, if the hen had laid an egg the morning of sampling days. In fact, individuals of both lines were selected for sampling if they laid an egg in the morning. The two lines were part of a larger study (Jansen et al., 2020a). Therefore, it was essential to keep the hens in single cages.

### Chicken Lines, Housing System, Management, and Feeding

The study is part of a comprehensive project (Dudde et al., 2018, 2020; Eusemann et al., 2018a, b, 2020; Krause and Schrader, 2018; Malchow et al., 2019; Jansen et al., 2020a, b). Within this project two high-performing purebred layer lines, BLA and WLA were used. BLA is a brown-egg strain originated from Rhode Island Red and New Hampshire and WLA is a white-egg layer line descended from White Leghorn. Both chicken lines go back to breeding lines of Lohmann Tierzucht GmbH (Cuxhaven, Germany) and have been maintained in a sire rotation program since 2012. Their average egg laying performance is about 320 eggs per year, the onset of laying is around the 21st week of age and reaches a maximum of 93% (BLA) to 96% (WLA) at 28 weeks of age (Lieboldt et al., 2015). For further information about performance data see Jansen et al. (2020a).

The experimental trial lasted from March 2017 to March 2018. From 15 weeks of age onward, 72 hens per layer line were kept in individual cages of a three-tiered system arranged in two rows separated by a grid in between. Each cage had a floor space of 2,400 cm^2^ and was equipped with a feeding through, a nipple drinker and a round plastic perch. Food and water were provided *ad libitum*. The lightning period was gradually stepped up from 9 h per day in the 15th week of age to 14 h per day (3.30 a.m. to 5.30 p.m.) at 21 weeks of age and remained constant until the end of the experimental trial.

### Data Collection

To determine the base value of blood parameters, first sampling was performed before the onset of lay in the 17th week of age. Further blood collection was made after the onset of lay in the 25th week of age, in week 29 close to the peak of the laying period, during laying persistency at 49 weeks and at the end of the laying period in week 69. Blood samples were taken during the light phase from 15 hens per layer line at 17 weeks and 10 to 12 hens per layer line within the following weeks of examination. Supplementary Table 1 provides an overview about the number of samples analysed for BLA- and WLA-hens within the single weeks of age and time points. On each sampling day, first sampling was made early in the morning starting at 6 a.m. shortly after oviposition. In each case, only hens that had just laid an egg were selected for the study. Further blood samples were drawn at 10 a.m., 2 p.m. and with the exception of the 17th week of age, at 6 p.m. Approximately 2 ml blood was drawn from the ulnar vein. Blood samples were collected in a 2-ml plastic syringe designed for blood gas analysis and containing dry lithium heparin (blood gas monovette) and K-EDTA-containing tubes (S-Monovette^®^ K3 EDTA; Sarstedt, Nümbrecht, Germany; 4 ml volume; final concentration 1.6 mg K-EDTA per ml whole blood). Immediately after sampling, ionized calcium (Ca2+) was determined using an automated blood gas analyser (RAPID-Lab^®^ 348- EX Blood Gas System, Siemens Diagnostic^TM^, Muenchen, Germany). EDTA-blood samples were swirled immediately and stored on ice. Within 2 h after venepuncture blood was centrifuged for 15 min at 4°C and 2,000 × *g* and plasma aliquots were pipetted in Eppendorf tubes, which were frozen at –80°C. Total calcium concentration was determined by EDTA titration using calcon carboxylic acid at pH > 12.0 according to the method described by Baczyk and Baranowska (1963). A commercial enzyme-linked immunosorbent assay (ELISA) kit (IBL International GmbH, Hamburg, Germany) was used to measure estradiol-17β in pg/ml. A pooled plasma sample was included on each kit together with the individual samples to calculate the inter-assay coefficient of variation which was 0.18. Each blood sample was measured in duplicate to calculate the intra-assay coefficient of variation. If the intra-assay coefficient was higher than ten, the measurement was repeated. CTX-I was evaluated using Serum CrossLaps ELISA (Immunodiagnostic Systems (IDS) Ltd, United Kingdom), IDS 25-Hydroxy Vitamin D EIA was used for determination of 25(OH)D_3_ and serum osteocalcin was measured by the commercial Quidel MicroVue Osteocalcin ELISA kit (Hanover, Germany) according to manufacturer’s instructions. A pooled plasma sample of ten laying hens (brown- and white-egg layers; approx. 300 μl plasma/hen) was analysed ten times and the mean value calculated was used as internal control for CTX-I, 25(OH)D_3_ and osteocalcin ELISAs, respectively. Moreover, each sample was measured twice together with the controls included in the ELISA kits. Measurements were repeated when the difference between two measurements of a sample was outside the range of variation of the internal controls. Plasma inorganic phosphate (PO4) concentrations were determined using the colorimetric molybdenum blue method (Armstrong et al., 1961).

### Statistical Analyses

Statistical analyses were performed using JMP, version 14.0 (Statistical Analysis System Institute, Cary, NC, United States).

Univariate analyses of variance (ANOVA *F* test) were used to examine whether means of blood parameters were significantly different from each other. If so, pairwise comparisons were made using the Tukey Kramer’s multiple comparison test. Results were regarded significant when the *P*-value was less than 0.05.

The following model was used to analyse data at 17 weeks of age:

$$Y_{ijk}=\mu +LL_{i}+T_{j}+LL*T_{ij}+e_{ijk}$$

where Y_*ijk*_ = observations of total calcium, ionized calcium, PO4, CTX-I, osteocalcin, 25(OH)D_3_ and estradiol-17β; μ = general mean; LL_*i*_ = fixed effect of layer line (i = 1 to 2); T_*j*_ = fixed effect of time of the day (j = 1 to 3); LL^∗^T_*ij*_ = interaction between the effects of layer line and time of the day and e_*ijk*_ = random error variance.

An extended model, including the effect of age and its interactions was used to analyse data from 25 to 69 weeks of age:

$$Y_{ijkl}=\mu +LL_{i}+T_{j}+A_{k}+LL*T_{ij}+LL*A_{ik}+T*A_{jk}+e_{ijkl}$$

where Y_*ijkl*_ = observations of total calcium, ionized calcium, PO4, CTX-I, osteocalcin, 25(OH)D_3_ and estradiol-17β; μ = general mean; LL_*i*_ = fixed effect of layer line (i = 1 to 2); T_*j*_ = fixed effect of time of the day (j = 1 to 4); A_*k*_ = fixed effect of age (k = 1 to 4); LL^∗^T_*ij*_ = interaction between the effects of layer line and time of the day; LL^∗^A_*ik*_ = interaction between the effects of layer line and age; T^∗^A_*jk*_ = interaction between the effects of time of the day and age and e_*ijkl*_ = random error variance.

The analyses of variance also included the three-way-interaction effect of layer line^∗^time of the day^∗^age, but due to non-significance this effect was excluded from the models.

A similar statistical model was used to compare concentrations of blood parameters measured in week 17 and 25, when the analyses included only data assessed at 6 a.m., 10 a.m. and 2 p.m. (Supplementary Table 4).

## Results

### Pre-laying Period (17 Weeks of Age)

Supplementary Table 2 shows the effects of the main factors and their interaction on blood concentrations of total and ionized calcium, PO4, CTX-I, osteocalcin, 25(OH)D_3_ and estradiol-17β at 17 weeks of age. The effect of layer line was significant for all parameters studied, except for ionized calcium and CTX-I. In contrast, time of day had no significant effect for most parameters, but was significant for CTX-I and PO4. The interaction of layer line and time of the day was not significant for the measured parameters, with the exception of CTX-I.

Across all times during the day, BLA hens had a significantly higher PO4 and osteocalcin concentration, but significantly lower total calcium, 25(OH)D_3_ and estradiol-17β values than WLA (Table 1). Within layer lines, significant differences between the three time points could only be detected for CTX-I measured in WLA, with highest levels shortly after oviposition (1.24 ng/ml), which significantly differed from values thereafter (0.92 ng/ml) (Supplementary Table 3).

**TABLE 1:** Least square means (LSM), their standard errors (SE) and significant differences between the effect levels for blood concentrations of total and ionized calcium, inorganic phosphate (PO4), the carboxyterminal crosslinked telopeptide of type I collagen (CTX-I), osteocalcin, 25-hydroxycholecalciferol (25(OH)D_3_) and estradiol-17β of the blood of brown-(BLA) and white-egg (WLA) laying hens examined in the 17th week of age.

Effect	Total calcium (mmol/l)	Ionized calcium (mmol/l)	PO4 (mmol/l)	CTX-I (ng/ml)	Osteocalcin (ng/ml)	25(OH)D_3_ (ng/ml)	Estradiol-17β (pg/ml)

	LSM ± SE	LSM ± SE	LSM ± SE	LSM ± SE	LSM ± SE	LSM ± SE	LSM ± SE
**Layer line**							
BLA	2.64^B^±0.06	1.37^A^±0.02	1.69^A^±0.04	1.00^A^±0.03	46.63^A^±4.33	47.10^B^±1.88	113.06^B^±18.93
WLA	2.83^A^±0.06	1.42^A^±0.02	1.36^B^±0.04	1.03^A^±0.03	35.48^B^±3.16	54.85^A^±1.88	237.60^A^±18.93
**Time of the day**							
6 a.m.	2.65^A^±0.07	1.39^A^±0.02	1.63^A^±0.04	1.15^A^±0.04	37.14^A^±4.27	51.03^A^±2.30	183.80^A^±23.18
10 a.m.	2.81^A^±0.07	1.37^A^±0.02	1.50^*AB*^±0.04	0.95^B^±0.04	39.62^A^±4.79	52.10^A^±2.30	177.37^A^±23.18
2 p.m.	2.74^A^±0.07	1.43^A^±0.02	1.45^B^±0.04	0.95^B^±0.04	46.42^A^±4.84	49.79^A^±2.30	164.82^A^±23.18

*^A,B^Means within a column and effect with no common superscript differ significantly at P < 0.05.*

Since blood sampling was only done at three time points during the day at 17 weeks of age, the analysis of variance of data assessed before and after the onset of lay, i.e., at 17 and 25 weeks of age, included only values assessed at 6 a.m., 10 a.m. and 2 p.m. As presented in Supplementary Table 4, among and within layer lines, the amount of CTX-I, osteocalcin and 25(OH)D_3_ measured before the onset of lay was significantly higher than the value detected after the onset of lay at 25 weeks of age. In contrast, in both layer lines the level of total calcium was significantly lower at 17 weeks of age compared to 25 weeks of age, while for estradiol-17β this was significant in the case of BLA only, but showed the same tendency for WLA. While across layer lines, levels of ionized calcium and PO4 assessed in the 17th week of age were significantly lower than those measured at 25 weeks of age, within layer lines these differences were only significant for BLA (ionized calcium) and WLA (PO4), respectively (Supplementary Table 4).

### Laying Period (25 to 69 Weeks of Age)

#### Effects of Layer Line and Age

Supplementary Table 5 shows the effects of the main factors and their interactions on blood concentrations of total and ionized calcium, PO4, CTX-I, osteocalcin, 25(OH)D_3_ and estradiol-17β at 25–69 weeks of age. The layer line had an effect on almost all blood parameters except PO4 and estradiol-17β, while the time of the day proved to be a significant factor for all parameters measured. The interaction of layer line and age was significant for concentration of total calcium, ionized calcium as well as 25(OH)D_3_. Results of analysis of variance for these traits are given in Table 2 and Supplementary Table 6, respectively.

**TABLE 2:** Least square means (LSM), their standard errors (SE) and significant differences between the levels of interaction effects for blood concentrations of total and ionized calcium, inorganic phosphate (PO4), the carboxyterminal crosslinked telopeptide of type I collagen (CTX-I), osteocalcin, 25-hydroxycholecalciferol (25(OH)D_3_) and estradiol-17β of the blood of brown-(BLA) and white-egg (WLA) laying hens examined in the 25th, 29th, 49th and 69th week of age.

Effect		Total calcium (mmol/l)	Ionized calcium (mmol/l)	PO4 (mmol/l)	CTX-I (ng/ml)	Osteocalcin (ng/ml)	25(OH)D_3_ (ng/ml)	Estradiol-17β (pg/ml)
		
		LSM ±SE	LSM ±SE	LSM ±SE	LSM ±SE	LSM ±SE	LSM ±SE	LSM ±SE
**Layer line × Time of the day**								
BLA	6 a.m.	6.37^AB^± 0.13	1.54^A^± 0.03	1.70^B^± 0.08	0.32^A^± 0.07	20.67^A^± 0.93	25.90^A^± 1.13	405.65^ABC^± 16.32
	10 a.m.	6.55^A^± 0.13	1.26^C^± 0.03	1.83^AB^± 0.08	0.28^A^± 0.07	15.39^B^± 0.92	25.77^A^± 1.13	405.50^ABC^± 16.32
	2 p.m.	6.33^AB^± 0.13	1.41^B^± 0.03	1.87^AB^± 0.08	0.13^A^± 0.07	13.94^BC^± 0.92	24.57^A^± 1.13	373.42^BC^± 16.32
	6 p.m.	5.87^BC^± 0.13	1.32^BC^± 0.03	1.97^AB^± 0.08	0.32^A^± 0.07	21.67^A^± 0.92	22.78^A^± 1.13	361.25^BC^± 16.32
WLA	6 a.m.	6.27^AB^± 0.13	1.44^AB^± 0.03	1.76^B^± 0.09	0.17^A^± 0.07	11.75^*BCD*^± 0.93	17.28^B^± 1.19	456.95^A^± 16.52
	10 a.m.	6.43^AB^± 0.13	1.21^C^± 0.03	1.70^B^± 0.09	0.09^A^± 0.07	9.65^*D*^± 0.95	17.11^B^± 1.19	414.12^AB^± 16.52
	2 p.m.	5.99^ABC^± 0.13	1.41^AB^± 0.03	1.62^B^± 0.09	0.07^A^± 0.07	8.77^*D*^± 0.93	16.30^B^± 1.19	396.49^ABC^± 16.52
	6 p.m.	5.64^C^± 0.13	1.27^C^± 0.03	2.17^A^± 0.09	0.31^A^± 0.07	11.19^*CD*^± 0.93	13.49^B^± 1.19	343.30^C^± 16.52
**Layer line × Age**								
BLA	25	4.95^*D*^± 0.13	1.45^AB^± 0.03	1.95^A^± 0.08	0.21^AB^± 0.07	20.73^A^± 0.98	28.64^A^± 1.25	257.87^F^±20.78
	29	5.84^C^± 0.13	1.21^C^± 0.03	1.65^A^± 0.08	0.45^A^± 0.07	18.10^AB^± 0.92	20.15^B^± 1.14	339.41^DE^±15.49
	49	7.10^A^± 0.13	1.51^A^± 0.03	1.88^A^± 0.08	0.13^B^± 0.07	16.18^B^± 0.92	20.57^B^± 1.25	415.34^BC^±14.70
	69	7.24^A^± 0.13	1.37^B^± 0.03	1.89^A^± 0.08	0.27^AB^± 0.07	16.63^AB^± 0.92	29.65^A^± 0.88	533.19^A^±14.70
WLA	25	4.80^*D*^± 0.14	1.44^AB^± 0.03	1.77^A^± 0.08	0.25^AB^± 0.07	14.31^B^± 1.05	16.50^BC^± 1.40	261.22^EF^±23.24
	29	5.84^C^± 0.13	1.05^C^± 0.03	1.74^A^± 0.08	0.18^AB^± 0.07	8.97^C^± 0.92	11.91^C^± 1.25	371.32^CD^±15.49
	49	6.43^B^± 0.13	1.44^AB^± 0.03	1.92^A^± 0.08	0.10^B^± 0.07	8.98^C^± 0.92	16.37^BC^± 1.25	457.68^B^±14.70
	69	7.25^A^± 0.12	1.42^AB^± 0.03	1.82^A^± 0.08	0.12^B^± 0.07	9.07^C^± 0.92	19.40^B^± 1.88	520.64^A^±13.42

*^A–F^Means within a column and effect with no common superscript differ significantly at P < 0.05.*

On average across all age groups and daily time points, the BLA line had significantly higher amounts of total calcium, ionized calcium, CTX-I, osteocalcin, and 25(OH)D_3_ than WLA, while the value of PO4 and estradiol-17β did not differ significantly between the layer lines. No significant effect of age on PO4 values was detected in BLA and WLA from 25 to 69 weeks of age. Concentrations of total calcium and estradiol-17β, however, increased with advancing age. Within layer lines significant differences for most parameters between all of the examined weeks have been detected, with the exception of total calcium in BLA hens, which was equal at 49 and 69 weeks of age. In contrast, osteocalcin levels were highest when hens came into lay (25 weeks of age) and significantly differed from levels detected thereafter in WLA, while in BLA a significant difference was only observed between the 25th and 49th week of age. Within and across layer lines lowest values of ionized calcium were measured when hens aged 29 weeks and significantly differed from those determined in the 25th, 49th and 69th week of age. Moreover, BLA hens had significantly lower Ca2+ levels at 69 than at 49 weeks of age. Across layer lines, and within BLA line, 25(OH)D_3_ concentrations measured at 25 and 69 weeks of age, i.e., shortly after the onset and at the end of the laying period, were significantly higher compared to values detected around the peak of laying (29 weeks of age) and the following persistency (49 weeks of age). In WLA, 25(OH)D_3_ levels were significantly lower in week 29 than in week 69, but did not differ significantly from the other weeks. No significant effect of age on CTX-I was detected in white-egg layers, but CTX-I levels measured for BLA hens at week 29 were more than three times higher (0.45 ng/ml) than those detected at week 49 (0.13 ng/ml).

#### Daily Egg-Laying Cycle

Within the laying period (25 to 69 weeks of age), 25(OH)D_3_ and estradiol-17β concentrations declined during the course of the day. This tendency occurred in BLA and WLA, but differences were significant only for the comparison of estradiol-17β levels measured in WLA in the morning (6 a.m. and 10 a.m.) and in the evening. However, considering the time course for each week separately (Supplementary Table 6), differences did not reach a significant level, with the exception of estradiol-17β measured in week 69, where the level was significantly higher at 6 a.m. compared to those determined at 10 a.m. Total calcium concentrations were almost equal from 6 a.m. to 2 p.m., with highest levels at 10 a.m. and significantly decreased to 6 p.m. Ionized calcium levels followed a sinusoidal pattern over the day with significantly higher levels detected in the early morning and afternoon in both layer lines. In contrast, PO4 levels detected at 6 a.m., 10 a.m. and 2 p.m. were lower than those measured in the evening, but significant differences could be shown only for WLA. Within single weeks of age, no significant differences between times of the day, but a tendency toward higher PO4 values in the evening could be seen (Supplementary Table 6). The overall effect of time of the day was significant for CTX-I, which decreased from 6 a.m. to 2 p.m. and increased thereafter with significant differences accounted between 2 p.m. and 6 p.m. However, the interaction effects of layer line and time of the day, and time of the day and age were not significant. Similar results have been obtained for osteocalcin, where higher levels were measured in the morning (6 a.m.) and evening (6 p.m.) and significantly differed from values detected at 10 a.m. and 2 p.m. in BLA.

## Discussion

### Effects of Layer Line and Age

We found clear differences related to the layer line when hens aged 17 weeks. In particular, the level of estradiol-17β calculated for WLA (237.6 pg/ml) was more than twice as high as the level calculated for BLA (113.1 pg/ml). In contrast, osteocalcin concentrations of BLA hens were significantly higher than those detected for WLA. These results are rather unexpected, as estrogens, including estradiol-17β, induce medullary bone formation (Bouvarel et al., 2011) from 16 weeks of age, when the ovary and oviduct are developing (Bain et al., 2016). Medullary bone serves as a labile source of calcium for the formation of eggshells (Whitehead, 2004). It is assumed that calcium in bone matrix is bound by osteocalcin, which is considered a marker of bone turnover based of its function of tightly coupling bone formation with resorption (Christenson, 1997). Due to these facts, we would have instead expected high estradiol-17β values and at the same time high osteocalcin levels, i.e., higher bone remodelling processes in WLA. During the following laying period, estradiol-17β values did not differ between BLA and WLA, whereas osteocalcin levels remained significantly higher in brown- than in white-egg layers. This suggests an increased bone turnover, including bone formation, possibly to cover the high demand of calcium for the formation of eggshells. Previous studies already detected a higher breaking strength and bone mineral density of the humerus (Habig et al., 2017; Jansen et al., 2020a) as well as a lower prevalence of keel bone deformities (Eusemann et al., 2018a) in BLA compared to WLA hens. During the pre-laying period, BLA hens had significantly lower 25(OH)D_3_ values than WLA, while in the course of the laying period the opposite was the case. In the cycle of mineral supply, 25(OH)D_3_ is converted to 1,25(OH)D_3_ in order to increase intestinal calcium absorption. Therefore, lower 25(OH)D_3_ blood concentrations measured in WLA from 25 to 69 weeks of age are supposed to be a result of a higher conversion rate of this vitamin D derivative. Nevertheless, 25(OH)D_3_ plasma levels mainly reflect the dietary supply of vitamin D (Nys and Le Roy, 2018) and the higher 25(OH)D_3_ values measured in BLA during the laying period might be caused by the higher feed consumption detected in BLA compared to WLA (Lieboldt et al., 2015; Jansen et al., 2020a). Taken together, our results suggest a higher calcium mobilisation from bone rather than from the diet in BLA hens, while WLA hens might cover the high calcium demands through gastrointestinal digestion. Together with previous observations on long bones (tibia and humerus) regarding lower bone mineral densities as well as lower humeral breaking strengths in WLA- compared to BLA-layers (Jansen et al., 2020a), our results raises questions, whether bone’s calcium reserves are exhausted in white-egg layers causing an increased, and maybe also more efficient calcium absorption from the diet. This is further supported by the fact that WLA hens have a significantly lower feed intake, but at the same time have a higher weight and proportion of egg shells, leading to a better feed-to-eggshell conversion ratio (Jansen et al., 2020a).

Estradiol-17β plasma concentrations rises sharply to reach a peak level 2–3 weeks before the first egg is laid (Senior, 1974). The higher levels of WLA hens measured at 17 weeks of age might be an indication that brown-egg layers were further from sexual maturity than white-egg layers. However, both lines laid their first egg at a mean age of 21 weeks (data not shown). Estradiol-17β plasma concentrations are supposed to be higher in egg laying hens compared to non-egg laying hens (Eusemann et al., 2018b), explaining the lower levels of 17-week-old hens compared to hens aged 25 weeks or older. The increase in estradiol-17β concentration on reaching sexual maturity was also described in a review by Nys and Le Roy (2018), although their data based on hitherto unpublished works. During the following laying period, estradiol-17β significantly increased from 25 weeks of age to a maximum at 69 weeks of age. Simultaneously, the concentration of total calcium increased rapidly from 17 to 25 weeks of age and steadily thereafter, which is in line with previous observations (Pavlík et al., 2009). In birds and other non-mammalian vertebrates, the regulation of hepatic vitellogenesis, i.e., production of egg yolk components, is mediated by estrogen, causing increased levels of total calcium in plasma (Simkiss, 1961) which may explain the unidirectional time course of estradiol-17β and total calcium levels in blood. According to Soares (1984), active laying hens have plasma calcium concentrations from 5 to 8.75 mmol/l, while non-layers have only half of these concentrations. Our results are in the range of these values and clearly show the difference between immature hens at 17 weeks of age (approx. 3 mmol/l) and egg-producing layers from 25 weeks of age onward (approx. 6 mmol/l). The range of total calcium levels as well as the significant increase measured at the beginning of the laying period is in accordance to Forte et al. (1983). In contrast to our results, they found decreasing levels of total calcium over the course of the laying period from 20 to 109 weeks of age, while in our study total calcium levels significantly increased from 25 to 69 weeks of age. As their investigation took place almost 40 years ago, the higher egg laying rate as well as prolonged laying persistency in today’s modern layer lines compared to previous generations might be one explanation of the observed differences to the results of the current study. The level of ionized calcium, by contrast, was lowest at 29 weeks of age, when hens reached peak production and consequently highest demands were placed on mineral metabolism.

In their review, Nys and Le Roy (2018) showed a steady decline of plasma osteocalcin from 5 to 25 weeks of age, which is in accordance to our findings, where osteocalcin levels measured in week 17 were up to twice as high as values detected in the 25th week of age. Jiang et al. (2013) reported a significant decrease of serum osteocalcin concentrations in Hy-line Brown laying hens kept in 2-bird cages from 63 to 75 weeks of age. Although we chose longer intervals between blood samplings and only week 69 fell within the time frame studied by Jiang et al. (2013), we couldn’t confirm their findings as osteocalcin levels were highest at the onset of lay (25 weeks of age), decreased until peak production (29 weeks of age) and remained almost constant until the end of the laying period.

### Daily Egg-Laying Cycle

Significant differences within a day were assessed for CTX-I levels of WLA hens when they were 17 weeks old and had not yet laid an egg (Supplementary Table 3). At this age hens’ body starts to prepare for the forthcoming laying period and medullary bone is already formed (Bain et al., 2016).

During the 24 h laying cycle, Kerschnitzki et al. (2014) examined the dynamics of microscopic and nanoscopic structural changes in medullary bone of commercial laying hens and monitored serum concentrations of ionized calcium, phosphate and PTH. In accordance to their findings, PO4 levels detected in the present study were highest in the evening, approximately 12 h after oviposition, which is in the middle of the stage of eggshell calcification and moreover represents the start of the phase, where most rapid rates of shell secretion occurs (12-18 h after oviposition) (Clunies and Leeson, 1995). As this phase takes place mainly at night, when no feed intake occurs, calcium is primarily mobilized from medullary bone. In the medullary bone, calcium is stored in form of hydroxyapatite crystals of calcium phosphate and it’s release simultaneously enhances phosphate concentrations in blood (Luck and Scanes, 1979; Kebreab et al., 2009; Kerschnitzki et al., 2014). While phosphate remains in the blood, because it is required for egg yolk synthesis (Kebreab et al., 2009) and not used for eggshell production, calcium is subsequently transported to the shell gland. Consequently, the amount of calcium in the blood decreases with progressive eggshell calcification (Sloan et al., 1974; Kerschnitzki et al., 2014). This may explain the observed decline of total and ionized calcium from 2 p.m. to 6 p.m., although not significant within layer lines. At the same time, an increased activity of the renal 25(OH)_2_-1-*α*-hydroxylase and elevated plasma 1,25(OH)D_3_ concentrations 14-16 h post-ovulation have been reported (Abe et al., 1979). As 1,25(OH)D_3_ is produced through hydroxylation of 25(OH)D_3_, latter might be diminished during the calcification process. Although within layer lines no significant differences between times of the day have been observed for 25(OH)D_3_ in the current study, the level tended to decrease over the course of the lightning period from 6 a.m. to 6 p.m. Former studies showed that the percentage of active osteoclasts as well as their resorption surface increases during the period of eggshell formation from 7 to 20 h after ovulation, resulting in an increase of the active resorption and active osteoblastic surfaces of medullary bone (van de Velde et al., 1984). These high bone turnover rates are reflected by the results of the current study, although not significant, with higher levels of CTX-I and osteocalcin measured in the evening, when shell formation is in full progress. Although a direct comparison between birds and mammals might be questionable, we measured higher levels of osteocalcin in the morning than in the afternoon, which is in accordance to dogs (Ladlow et al., 2002).

To the best of our knowledge, this is the latest study examining bone-associated blood parameters over the course of the egg laying period and the daily egg laying cycle in two phylogenetically divergent layer lines. It has been revealed by our study, that in the course of the light period, from 6 a.m. to 6 p.m., blood concentrations of total and ionized calcium, PO4, CTX-I, osteocalcin, 25(OH)D_3_ and estradiol-17β are subjected to no or only slight variations and clearly reflect the period of the daily egg laying cycle. The age of laying hens and corresponding stage of egg laying, however, had a significant effect on most of the examined parameters. Moreover, differences between brown- and, white-egg layers with a similar performance level raise questions, whether their phylogenetic background determines their efficiency to cope with high demands on calcium metabolism related to egg production. When interpreting the significant results, however, it must be remembered that these are based on univariate analyses, i.e., each trait is considered separately. Further research in the field of resource allocation is needed to get insides into the complex processes of mineral metabolism in laying hens of different origin.

## Data Availability Statement

The raw data supporting the conclusions of this article will be made available by the authors, without undue reservation.

## Ethics Statement

The current experiment was performed in accordance with the German Animal Welfare Law and approved by the Lower Saxony State Office for Consumer Protection and Food Safety (33.19-42502-04-15/1988).

## Author Contributions

CH, AW, and SW: conceptualisation, sampling. SW: funding acquisition, supervision. AW and SP: laboratory analysis. CH and UB: statistical analyses. CH: formal analysis, writing – original draft preparation. CH, AW, UB, SP, and SW: writing – review and editing. All authors have read and agreed to the published version of this manuscript.

## Conflict of Interest

The authors declare that the research was conducted in the absence of any commercial or financial relationships that could be construed as a potential conflict of interest.
