# Supplementary material for: Influence of Age and Phylogenetic Background on Blood Parameters Associated With Bone Metabolism in Laying Hens
Source: Front Physiol. 2021 Apr 29;12:678054. doi: 10.3389/fphys.2021.678054 (PMC8117343; doi:10.3389/fphys.2021.678054)
Supplement: Supplementary file 2 [file Table_1.docx]

Supplementary Table 1. Number of examined hens

| Trait | Age (weeks) | 17 | | 25 | | 29 | | 49 | | 69 | |
| --- | --- | --- | --- | --- | --- | --- | --- | --- | --- | --- | --- |
|  | Layer line | BLA | WLA | BLA | WLA | BLA | WLA | BLA | WLA | BLA | WLA |
|  | Time |  |  |  |  |  |  |  |  |  |  |
| Total calcium | 6 a.m. | 15 | 15 | 10 | 9 | 10 | 10 | 10 | 10 | 10 | 12 |
|  | 10 a.m. | 15 | 15 | 10 | 9 | 10 | 10 | 10 | 10 | 10 | 12 |
|  | 2 p.m. | 15 | 15 | 10 | 9 | 10 | 10 | 10 | 10 | 10 | 12 |
|  | 6 p.m. | / | / | 10 | 9 | 10 | 10 | 10 | 10 | 10 | 12 |
| Ionized calcium | 6 a.m. | 13 | 13 | 10 | 9 | 10 | 10 | 10 | 10 | 10 | 12 |
|  | 10 a.m. | 15 | 14 | 9 | 9 | 10 | 10 | 10 | 10 | 10 | 12 |
|  | 2 p.m. | 15 | 13 | 10 | 9 | 10 | 10 | 10 | 10 | 10 | 12 |
|  | 6 p.m. | / | / | 10 | 9 | 10 | 10 | 10 | 10 | 10 | 12 |
| PO4 | 6 a.m. | 10 | 10 | 10 | 8 | 10 | 10 | 10 | 9 | 10 | 10 |
|  | 10 a.m. | 10 | 10 | 10 | 8 | 10 | 10 | 10 | 9 | 10 | 10 |
|  | 2 p.m. | 10 | 10 | 10 | 8 | 10 | 10 | 10 | 9 | 10 | 10 |
|  | 6 p.m. | / | / | 10 | 8 | 10 | 10 | 10 | 9 | 10 | 10 |
| CTX-I | 6 a.m. | 15 | 15 | 10 | 9 | 10 | 10 | 10 | 10 | 10 | 10 |
|  | 10 a.m. | 15 | 15 | 10 | 9 | 10 | 10 | 10 | 10 | 10 | 10 |
|  | 2 p.m. | 15 | 15 | 10 | 9 | 10 | 10 | 10 | 10 | 10 | 10 |
|  | 6 p.m. | / | / | 10 | 9 | 10 | 10 | 10 | 10 | 10 | 10 |
| Osteocalcin | 6 a.m. | 10 | 14 | 8 | 8 | 10 | 10 | 10 | 9 | 10 | 10 |
|  | 10 a.m. | 7 | 13 | 9 | 7 | 10 | 10 | 10 | 9 | 10 | 10 |
|  | 2 p.m. | 7 | 13 | 9 | 8 | 10 | 10 | 10 | 9 | 10 | 10 |
|  | 6 p.m. | / | / | 9 | 8 | 10 | 10 | 10 | 9 | 10 | 10 |
| 25(OH)D_3_ | 6 a.m. | 10 | 10 | 5 | 4 | 6 | 5 | 5 | 5 | 10 | 10 |
|  | 10 a.m. | 10 | 10 | 5 | 4 | 6 | 5 | 5 | 5 | 10 | 10 |
|  | 2 p.m. | 10 | 10 | 5 | 4 | 6 | 5 | 5 | 5 | 10 | 10 |
|  | 6 p.m. | / | / | 5 | 4 | 6 | 5 | 5 | 5 | 10 | 10 |
| Estradiol-17β | 6 a.m. | 5 | 5 | 5 | 4 | 9 | 9 | 10 | 10 | 10 | 12 |
|  | 10 a.m. | 5 | 5 | 5 | 4 | 9 | 9 | 10 | 10 | 10 | 12 |
|  | 2 p.m. | 5 | 5 | 5 | 4 | 9 | 9 | 10 | 10 | 10 | 12 |
|  | 6 p.m. | / | / | 5 | 4 | 9 | 9 | 10 | 10 | 10 | 12 |
