# Supplementary material for: Influence of Age and Phylogenetic Background on Blood Parameters Associated With Bone Metabolism in Laying Hens
Source: Front Physiol. 2021 Apr 29;12:678054. doi: 10.3389/fphys.2021.678054 (PMC8117343; doi:10.3389/fphys.2021.678054)
Supplement: Supplementary file 3 [file Table_2.docx]

Supplementary Table 2. The effects of layer line, time of the day and layer line x time of the day on blood concentrations of total and ionized calcium, inorganic phosphate (PO4), the carboxyterminal crosslinked telopeptide of type I collagen (CTX-I), osteocalcin, 25-hydroxycholecalciferol (25(OH)D_3_) and estradiol‑17β at 17 weeks of age.

| **Effect** | Total calcium  [mmol/l] | | Ionized calcium  [mmol/l] | | PO4  [mmol/l] | | CTX-I  [ng/ml] | | Osteocalcin  [ng/ml] | | 25(OH)D_3_  [ng/ml] | | Estradiol-17β  [pg/ml] | |
| --- | --- | --- | --- | --- | --- | --- | --- | --- | --- | --- | --- | --- | --- | --- |
|  | *F* Value | *p*- Value | *F* Value | *p*- Value | *F* Value | *p*- Value | *F* Value | *p*- Value | *F* Value | *p*- Value | *F* Value | *p*- Value | *F* Value | *p*- Value |
| Layer line (LL) | 5.28 | 0.0241 | 3.66 | 0.0594 | 42.17 | <0.0001 | 0.21 | 0.6450 | 4.33 | 0.0416 | 8.52 | 0.0051 | 21.65 | 0.0001 |
| Time of the day (T) | 1.30 | 0.2772 | 2.01 | 0.1403 | 4.64 | 0.0139 | 7.71 | 0.0008 | 1.07 | 0.3488 | 0.25 | 0.7772 | 0.17 | 0.8419 |
| LL x T | 0.45 | 0.6417 | 0.88 | 0.4201 | 0.50 | 0.6120 | 3.27 | 0.0429 | 0.21 | 0.8130 | 0.77 | 0.4680 | 0.34 | 0.7132 |
