# Supplementary material for: Influence of Age and Phylogenetic Background on Blood Parameters Associated With Bone Metabolism in Laying Hens
Source: Front Physiol. 2021 Apr 29;12:678054. doi: 10.3389/fphys.2021.678054 (PMC8117343; doi:10.3389/fphys.2021.678054)
Supplement: Supplementary file 6 [file Table_5.docx]

Supplementary Table 5. The effects of layer line, time of the day, age, layer line x time of the day, layer line x age and time of the day x age on blood concentrations of total and ionized calcium, inorganic phosphate (PO4), the carboxyterminal crosslinked telopeptide of type I collagen (CTX‑I), osteocalcin, 25-hydroxycholecalciferol (25(OH)D_3_) and estradiol‑17β from 25 to 69 weeks of age.

| **Effect** | Total calcium  [mmol/l] | | Ionized calcium  [mmol/l] | | PO4  [mmol/l] | | CTX-I  [ng/ml] | | Osteocalcin  [ng/ml] | | 25(OH)D_3_  [ng/ml] | | Estradiol-17β  [pg/ml] | | |
| --- | --- | --- | --- | --- | --- | --- | --- | --- | --- | --- | --- | --- | --- | --- | --- |
|  | *F* Value | *p*- Value | *F* Value | *p*- Value | *F* Value | *p*- Value | *F* Value | *p*- Value | *F* Value | *p*- Value | *F* Value | *p*- Value | *F* Value | *p*- Value |  |
| Layer line (LL) | 4.65 | 0.0318 | 4.72 | 0.0306 | 0.22 | 0.6379 | 3.99 | 0.0468 | 132.34 | <0.0001 | 107.78 | <0.0001 | 1.87 | 0.1727 |  |
| Time of the day (T) | 11.46 | <0.0001 | 29.25 | <0.0001 | 7.42 | <0.0001 | 3.13 | 0.0261 | 15.36 | <0.0001 | 3.62 | 0.0142 | 8.22 | <0.0001 |  |
| Age (A) | 125.51 | <0.0001 | 55.15 | <0.0001 | 2.39 | 0.0687 | 2.56 | 0.0555 | 11.24 | <0.0001 | 25.32 | <0.0001 | 86.92 | <0.0001 |  |
| LL x T | 0.34 | 0.7996 | 0.97 | 0.4083 | 2.84 | 0.0383 | 0.64 | 0.5869 | 3.77 | 0.0112 | 0.07 | 0.9757 | 1.67 | 0.1736 |  |
| LL x A | 2.99 | 0.0314 | 4.79 | 0.0028 | 1.01 | 0.3889 | 1.81 | 0.1459 | 0.74 | 0.5268 | 3.73 | 0.0124 | 1.46 | 0.2259 |  |
| T x A | 0.51 | 0.8683 | 23.02 | <0.0001 | 0.77 | 0.6487 | 1.24 | 0.2703 | 1.24 | 0.2697 | 0.33 | 0.9632 | 2.64 | 0.0061 |  |
