# Supplementary material for: Influence of Age and Phylogenetic Background on Blood Parameters Associated With Bone Metabolism in Laying Hens
Source: Front Physiol. 2021 Apr 29;12:678054. doi: 10.3389/fphys.2021.678054 (PMC8117343; doi:10.3389/fphys.2021.678054)
Supplement: Supplementary file 7 [file Table_6.docx]

Supplementary Table 6. Least square means (LSM), their standard errors (SE) and significant differences between the effect levels for blood concentrations of total and ionized calcium, inorganic phosphate (PO4), the carboxyterminal crosslinked telopeptide of type I collagen (CTX-I), osteocalcin, 25-hydroxycholecalciferol (25(OH)D_3_) and estradiol-17β of the blood of brown-(BLA) and white-egg (WLA) laying hens examined in the 25^th^, 29^th^, 49^th^ and 69^th^ week of age.

| **Effect** | Total calcium  [mmol/l] | | Ionized calcium  [mmol/l] | | PO4  [mmol/l] | | CTX-I  [ng/ml] | | Osteocalcin  [ng/ml] | | 25(OH)D_3_  [ng/ml] | | Estradiol-17β  [pg/ml] | |
| --- | --- | --- | --- | --- | --- | --- | --- | --- | --- | --- | --- | --- | --- | --- |
|  | LSM ± SE | | LSM ± SE | | LSM ± SE | | LSM ± SE | | LSM ± SE | | LSM ± SE | | LSM ± SE | |
| **Layer line** | | | | | | | | | | | | | | |
| BLA | 6.28^A^ ± | 0.07 | 1.38^A^ ± | 0.02 | 1.84^A^ ± | 0.04 | 0.26^A^ ± | 0.04 | 17.91^A^ ± | 0.47 | 24.75^A^ ± | 0.57 | 386.45^A^ ± | 8.31 |
| WLA | 6.08^B^ ± | 0.07 | 1.34^B^ ± | 0.01 | 1.81^A^ ± | 0.04 | 0.16^B^ ± | 0.04 | 10.33^B^ ± | 0.47 | 16.05^B^ ± | 0.61 | 402.72^A^ ± | 8.57 |
| **Time of the day** | | | | | | | | | | | | | | |
| 6 a.m. | 6.32^A^ ± | 0.09 | 1.49^A^ ± | 0.02 | 1.73^B^ ± | 0.06 | 0.24^AB^ ± | 0.05 | 16.21^A^ ± | 0.66 | 21.59^A^ ± | 0.84 | 431.30^A^ ± | 11.86 |
| 10 a.m. | 6.49^A^ ± | 0.09 | 1.24^C^ ± | 0.02 | 1.76^B^ ± | 0.06 | 0.19^AB^ ± | 0.05 | 12.52^B^ ± | 0.66 | 21.44^A^ ± | 0.84 | 409.81^AB^ ± | 11.86 |
| 2 p.m. | 6.16^A^ ± | 0.09 | 1.41^B^ ± | 0.02 | 1.75^B^ ± | 0.06 | 0.10^B^ ± | 0.05 | 11.35^B^ ± | 0.66 | 20.43^AB^ ± | 0.84 | 384.96^BC^ ± | 11.86 |
| 6 p.m. | 5.75^B^ ± | 0.09 | 1.30^C^ ± | 0.02 | 2.07^A^ ± | 0.06 | 0.32^A^ ± | 0.05 | 16.43^A^ ± | 0.66 | 18.14^B^ ± | 0.84 | 352.28^C^ ± | 11.86 |
| **Age (weeks)** | | | | | | | | | | | | | | |
| 25 | 4.88^D^ ± | 0.10 | 1.44^AB^ ± | 0.02 | 1.86^A^ ± | 0.06 | 0.23^AB^ ± | 0.05 | 17.52^A^ ± | 0.72 | 22.57^A^ ± | 0.94 | 259.55^D^ ± | 15.59 |
| 29 | 5.84^C^ ± | 0.09 | 1.13^C^ ± | 0.02 | 1.69^A^ ± | 0.06 | 0.31^A^ ± | 0.05 | 13.53^B^ ± | 0.65 | 16.03^B^ ± | 0.85 | 355.36^C^ ± | 10.95 |
| 49 | 6.77^B^ ± | 0.09 | 1.48^A^ ± | 0.02 | 1.90^A^ ± | 0.06 | 0.12^B^ ± | 0.05 | 12.58^B^ ± | 0.65 | 18.47^B^ ± | 0.88 | 436.51^B^ ± | 10.39 |
| 69 | 7.24^A^ ± | 0.09 | 1.39^B^ ± | 0.02 | 1.85^A^ ± | 0.06 | 0.19^AB^ ± | 0.05 | 12.85^B^ ± | 0.65 | 24.52^A^ ± | 0.63 | 526.92^A^ ± | 9.95 |

^A-D^ Means within a column and effect with no common superscript differ significantly at P < 0.05.

Supplementary Table 6. continued

| **Effect** | | Total calcium  [mmol/l] | | Ionized calcium  [mmol/l] | | PO4  [mmol/l] | | CTX-I  [ng/ml] | | Osteocalcin  [ng/ml] | | 25(OH)D_3_  [ng/ml] | | Estradiol-17β  [pg/ml] | |
| --- | --- | --- | --- | --- | --- | --- | --- | --- | --- | --- | --- | --- | --- | --- | --- |
|  | | LSM ± SE | | LSM ± SE | | LSM ± SE | | LSM ± SE | | LSM ± SE | | LSM ± SE | | LSM ± SE | |
| **Time of the day x Age** | | | | | | | | | | | | | | | |
| 6 a.m. | 25 | 5.21^FGH^ ± | 0.19 | 1.59^AB^ ± | 0.04 | 1.94^AB^ ± | 0.12 | 0.20^A^ ± | 0.10 | 20.49^AB^ ± | 1.45 | 25.51^AB^ ± | 1.87 | 277.48^GHI^ ± | 30.93 |
|  | 29 | 5.96^DEF^ ± | 0.19 | 1.44^ABCD^ ± | 0.04 | 1.48^B^ ± | 0.12 | 0.43^A^ ± | 0.10 | 15.07^ABCD^ ± | 1.30 | 16.19^CD^ ± | 1.69 | 387.94^DEFGH^ ± | 21.82 |
|  | 49 | 6.81^ABCD^ ± | 0.19 | 1.56^AB^ ± | 0.04 | 1.85^AB^ ± | 0.12 | 0.14^A^ ± | 0.10 | 15.91^ABCD^ ± | 1.30 | 18.70^ABCD^ ± | 1.77 | 462.69^BCDE^ ± | 20.70 |
|  | 69 | 7.29^A^ ± | 0.18 | 1.39^BCDEF^ ± | 0.04 | 1.65^AB^ ± | 0.12 | 0.20^A^ ± | 0.10 | 13.37^CD^ ± | 1.30 | 25.95^A^ ± | 1.25 | 597.07^A^ ± | 19.78 |
| 10 a.m | 25 | 5.20^FGH^ ± | 0.19 | 1.51^ABC^ ± | 0.05 | 1.69^AB^ ± | 0.12 | 0.08^A^ ± | 0.10 | 16.08^ABCD^ ± | 1.46 | 23.77^ABC^ ± | 1.87 | 331.36^FGHI^ ± | 30.93 |
|  | 29 | 6.08^CDEF^ ± | 0.19 | 0.64^H^ ± | 0.04 | 1.65^AB^ ± | 0.12 | 0.39^A^ ± | 0.10 | 11.63^CD^ ± | 1.30 | 16.49^CD^ ± | 1.69 | 364.93^EFGH^ ± | 21.82 |
|  | 49 | 7.05^AB^ ± | 0.19 | 1.61^A^ ± | 0.04 | 1.86^AB^ ± | 0.12 | 0.09^A^ ± | 0.10 | 10.61^CD^ ± | 1.30 | 19.93^ABCD^ ± | 1.77 | 458.49^BCDEF^ ± | 20.70 |
|  | 69 | 7.63^A^ ± | 0.19 | 1.19^G^ ± | 0.04 | 1.85^AB^ ± | 0.12 | 0.19^A^ ± | 0.10 | 11.63^CD^ ± | 1.30 | 25.57^A^ ± | 1.25 | 484.47^BCD^ ± | 19.78 |
| 2 p.m. | 25 | 4.73^GH^ ± | 0.19 | 1.39^BCDEFG^ ± | 0.04 | 1.78^AB^ ± | 0.12 | 0.09^A^ ± | 0.10 | 12.55^CD^ ± | 1.41 | 21.97^ABCD^ ± | 1.87 | 230.43^I^ ± | 30.93 |
|  | 29 | 5.79^EF^ ± | 0.19 | 1.22^EFG^ ± | 0.04 | 1.70^AB^ ± | 0.12 | 0.10^A^ ± | 0.10 | 10.48^D^ ± | 1.30 | 17.00^BCD^ ± | 1.69 | 383.09^DEFGH^ ± | 21.82 |
|  | 49 | 6.95^ABC^ ± | 0.19 | 1.48^ABC^ ± | 0.04 | 1.78^AB^ ± | 0.12 | 0.08^A^ ± | 0.10 | 10.14^D^ ± | 1.30 | 18.61^ABCD^ ± | 1.77 | 421.93^CDEF^ ± | 20.70 |
|  | 69 | 7.17^A^ ± | 0.18 | 1.57^AB^ ± | 0.04 | 1.72^AB^ ± | 0.12 | 0.15^A^ ± | 0.10 | 12.18^CD^ ± | 1.30 | 24.11^AB^ ± | 1.25 | 504.38^ABC^ ± | 19.78 |
| 6 p.m | 25 | 4.36^H^ ± | 0.19 | 1.30^CDEFG^ ± | 0.04 | 2.05^AB^ ± | 0.12 | 0.54^A^ ± | 0.10 | 20.97^A^ ± | 1.41 | 19.04^ABCD^ ± | 1.87 | 261.22^EF^ ± | 23.24 |
|  | 29 | 5.53^EFG^ ± | 0.19 | 1.21^FG^ ± | 0.04 | 1.94^AB^ ± | 0.12 | 0.33^A^ ± | 0.10 | 16.96^ABC^ ± | 1.30 | 14.44^D^ ± | 1.69 | 371.32^CD^ ± | 15.49 |
|  | 49 | 6.25^BCDE^ ± | 0.19 | 1.26^DEFG^ ± | 0.04 | 2.10^A^ ± | 0.12 | 0.16^A^ ± | 0.10 | 13.65^CD^ ± | 1.30 | 16.66^BCD^ ± | 1.77 | 457.68^B^ ± | 14.70 |
|  | 69 | 6.87^ABC^ ± | 0.18 | 1.42^ABCDE^ ± | 0.04 | 2.20^A^ ± | 0.12 | 0.23^A^ ± | 0.10 | 14.23^BCD^ ± | 1.30 | 22.47^ABC^ ± | 1.25 | 520.64^A^ ± | 13.42 |

^A-I^ Means within a column and effect with no common superscript differ significantly at P < 0.05.
